# Addressing the Precipitation of Hydrated Carbonates on a Bronze Cannon from the Alamo

**DOI:** 10.1021/acsomega.5c03779

**Published:** 2025-08-19

**Authors:** Kimberly L. Breyfogle, Christopher Dostal

**Affiliations:** Department of Anthropology, 14736 Texas A&M University, College Station, Texas United States

## Abstract

Following their defeat in the Texas Revolution of 1836, the Mexican Army disabled and buried cannons used in the defense of the Alamo. Rediscovered in 1852, 13 of these cannons have since journeyed through private collections and public exhibits before arriving at the Alamo. Among them is a bronze 4-pounder cannon, thought to have seen action during the battle itself. In 2017, the gun was conserved using electrolytic reduction with sodium hydroxide. White powder later appeared around the breech, identified as thermonatrite (Na_2_CO_3_·H_2_O), trona (Na_3_H­(CO_3_)_2_·2H_2_O), and spertiniite (Cu­(OH)_2_). The precipitate is likely the side effect of conservation treatment, and the cannon was retreated and boiled in deionized water in an attempt to remove all carbonates. This was ineffective, and an experiment was conducted to determine and effective way to neutralize the carbonates. Formic and citric acids were found to have the least negative effects on the experimental ingots, while phosphoric, acetic, and sulfuric acids were ruled out as too problematic. Formic acid was the most effective at preventing recurring precipitation, and was chosen for application to the artifact. It successfully kept the precipitate at bay for three months before reapplication was required, which was expected based on the experiment. Time to reapplication is expected to lengthen as the carbonate reacts with the formic acid and is removed.

## Graphical abstract

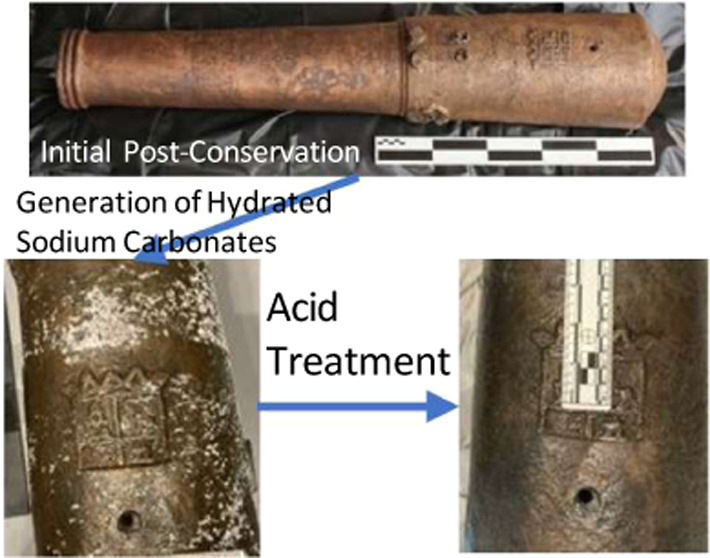

## Introduction

In 2017, the Conservation Research Laboratory (CRL) at Texas A&M University partnered with the Alamo Trust to conserve several iron and bronze cannons in the Alamo’s collection by removing paint from the surface of the guns, conserving them through electrolysis, and applying a new protective coating to the metal surface. The cannons had been on display outside for over 50 years without conservation. Among the cannons conserved was one bronze 4-pounder cannon shown in Figure 1. The cannon, which had a Spanish crest on its breech, was missing its trunnions, dolphins, and cascabel when it was brought for conservation.

**Figure 1:**
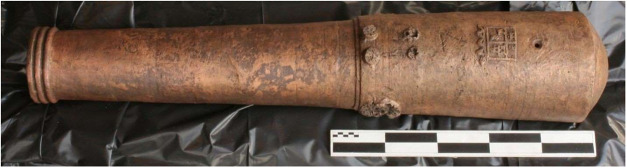
Bronze 4-pounder cannon immediately after conservation in 2017 (Photo credit: Conservation Research Laboratory, Texas A&M University).

### The Cannon

The Spanish crest on the breech is a clear indicator that the cannon is of Spanish origin. By tracing cannon inventories and movement through the region, this cannon has been linked to 12 cannons ordered by the governor of Los Adaes for protection in the early 1760s.^1^ The cannons were approved in 1763, and 12 4-pounders were dispatched to the presidio for protection.^2^

After Los Adaes was abandoned as the capital of Texas in 1773, this bronze cannon collection was transferred to San Antonio.^1^ Following numerous transfers, one bronze 4- pounder from the 1763 lot was used alongside other artillery by the Texian forces as they defended the Alamo, a small Spanish mission converted to a fort, against the Mexican Army. After the roughly 200 Texian soldiers were overwhelmed and killed by the Mexican forces, the Mexican commanders seized, destroyed, and buried this cannon along with the others.^3,4^

Sixteen years later in 1852, 13 mutilated cannons were discovered during an excavation to build a fence on a property near the Alamo.^5^ These cannons had been spiked and had their trunnions and cascabels broken off, indicating that they were likely the same cannons destroyed and buried by the Mexican Army. Among them were four bronze and nine iron cannons.

One of the bronze cannons, a 4-pounder, was reportedly gifted by the owner of the property, Samuel Maverick, to a friend, who then sent it to Philadelphia, PA.^3^ The cannon changed hands several more times before being donated to the San Jacinto Battleground Conservancy (SJBC), which then loaned the gun to the Alamo.^6^ It was sent to the CRL for conservation treatment in 2008 prior to being displayed at the Alamo. Because of a lack of documentation on the transfer between Maverick and the family in Pennsylvania, it is not entirely certain that the gun is the same one which was buried after the Texas Revolution. However, the destruction of the trunnions and cascabel, the form of the gun, and family history attesting to its origins support a positive identification of the gun as one of the bronze 4-pounder Alamo guns.

### Conservation

In 2008, the cannon was conserved using electrolytic reduction in a solution of 2% w/w sodium hydroxide (NaOH) in deionized water. Treatment occurred in a mild steel vat which was used as the main anode. Following treatment, it was boiled in deionized water until the pH stabilized at 7 to ensure that all the sodium hydroxide had been removed, mechanically polished using polishing brushes, soaked in a solution of 2% benzotriazole (BTA) and ethanol (w/w), and finally coated with microcrystalline wax. To coat with microcrystalline wax, the cannon was placed in molten wax until bubbles ceased forming, indicating the absence of any volatile solvent remaining in the matrix. The initial results were excellent, and the gun was displayed prominently until it was sent back to the CRL for reconservation in 2017 along with a large batch of cast iron cannons. There were some small patches of green patina that had formed on the gun in the intervening years, and it was the preference of the Alamo curation team to display the gun free of any corrosion or patina.

Upon arrival in 2017, the cannon was first boiled in hot deionized water to remove the coating of microcrystalline wax, then it underwent an identical process of electrolysis for 35 days in 2% w/w sodium hydroxide (NaOH) in deionized water as outlined above. Electrical current was kept at 5 A and 5 V, which is standard procedure for electrolysis at the CRL. It was boiled in three boiling water rinses over a period of 3 weeks. Immersion in the benzotriazole solution lasted 24 h. Conservation was completed and the gun was returned to the Alamo with the cast iron guns in early 2021.

Shortly before the gun was set to be sent back to the Alamo, a white powder began to precipitate on the cannon’s surface outside the microcrystalline wax coating, which is visible on the breech in Figure 2. The powder was concentrated primarily on the breech of the cannon, and was visible both on the surface of the exterior and inside the bore. It was agreed that the cannon would return to the CRL to address this powder after a short period of display for several planned events at the Alamo. A topical solution of 3% citric acid was sent with the curation team to temporarily neutralize the precipitate for events before analysis and reconservation could commence.

**Figure 2:**
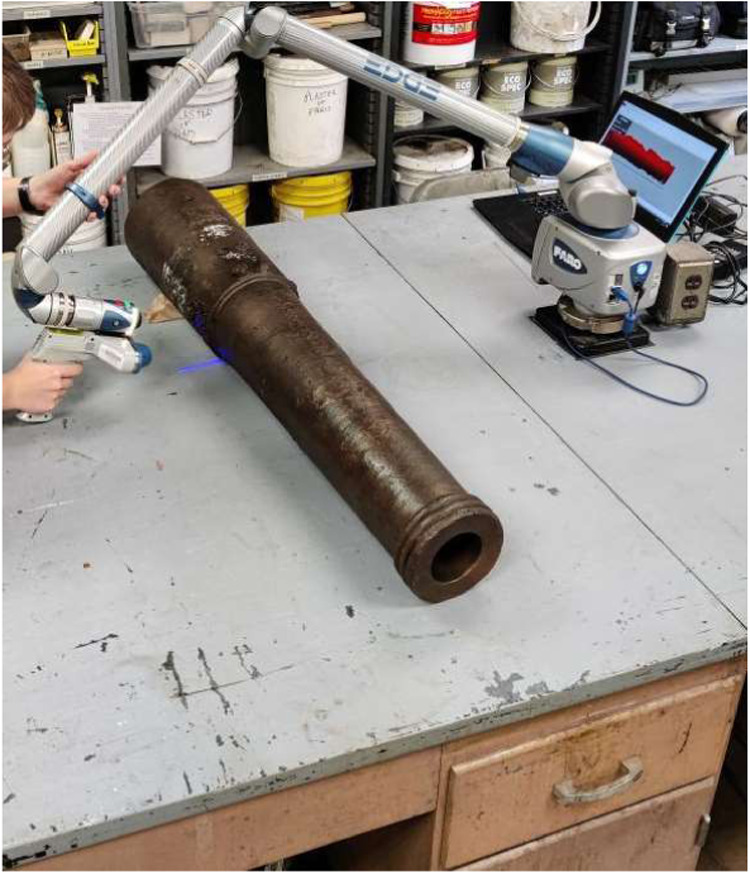
White precipitate visible on the breech of the cannon in January of 2021 as the gun was laser scanned shortly before returning to the Alamo.

The cannon was returned to the CRL for analysis and reconservation in 2022. After taking a sample of the powder, the wax was removed and the cannon was retreated with another round of electrolytic reduction for 149 days in 2% NaOH at 5 A and 5 V, boiled over a period of 3 weeks until the pH stabilized, mechanically polished using brushes, treated with 2% (w/w) BTA in ethanol, and coated in microcrystalline wax. During this reconservation period, the cannon was allowed to soak for 1 week in BTA. Almost immediately after the second round of conservation, the precipitate reformed. Subsequently, the cannon was treated with electrolytic reduction in 2% sodium carbonate (Na_2_CO_3_) at 5 A and 5 V, followed by extended boiling rinses in deionized water beyond the point at which the pH stabilized, 2% BTA treatment, and a microcrystalline wax coating. The precipitate returned after the first treatment in Na_2_CO_3_, so it was repeated. This time, the cannon was kept under cathodic protection during the boiling deionized water rinses. The precipitate has continued to return as seen in Figure 3, despite continued conservation efforts, including the application of low concentration citric acid to the cannon’s surface. As time progressed, green copper corrosion products began appearing alongside the white precipitate.

**Figure 3:**
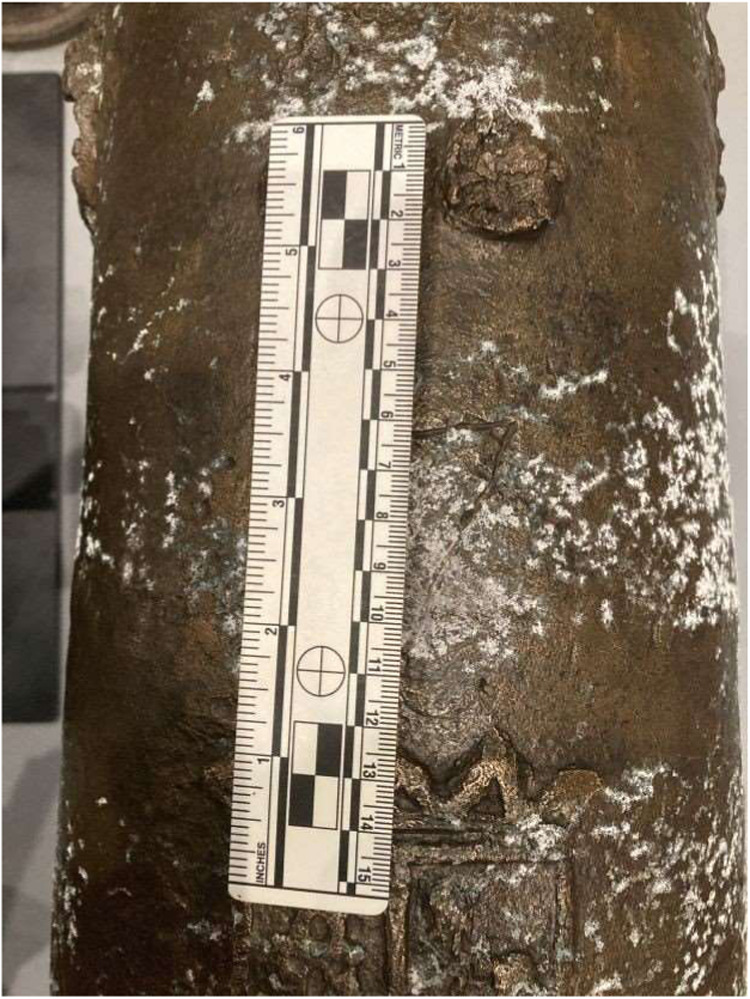
A white precipitate appeared on the surface of an historic bronze cannon. Traces of green copper corrosion are also visible on the surface (center, below scale bar).

A summary of the conservation parameters for each iteration is provided in Table 1.

**Table 1:** Summary of Conservation Parameters and Results

year	electrolyte	treatment time (if known)	voltage (V)	amperage (A)	preservative coating	protective coating	outcome
2008	2% NaOH in deionized H_2_O	--	5	5	2% BTA in EtOH	micro- crystalline wax	excellent, small patches of corrosion/patina after 9 years
2017	2% NaOH in deionized H_2_O	35 days	5	5	2% BTA in EtOH	micro- crystalline wax	white precipitate after a few months
2021	2% NaOH in deionized H_2_O	149 days	5	5	2% BTA in EtOH	micro- crystalline wax	white precipitate after a few days
2022	2% Na_2_CO_3_ in deionized H_2_O	--	5	5	2% BTA in EtOH	micro- crystalline wax	white precipitate after a few days
2022	2% Na_2_CO_3_ in deionized H_2_O	--	5	5	2% BTA in EtOH	micro- crystalline wax	white precipitate after a few days

### Powder Identification

In March 2022, a sample of the white powder was removed from the breech of the cannon and tested using X-ray diffraction (XRD) at the Department of Chemistry at Texas A&M University (Instrument specifications and methodology are provided in the Supporting Information). These initial results shown in Figure 4 revealed the presence of a high quantity of two hydrated sodium carbonate varieties: thermonatrite (a hydrated sodium carbonate salt, Na_2_CO_3_·H_2_O) and trona (also called sodium sesquicarbonate, Na_3_H­(CO_3_)_2_·2H_2_O), along with spertiniite (copper hydroxide, CuO_2_).

**Figure 4:**
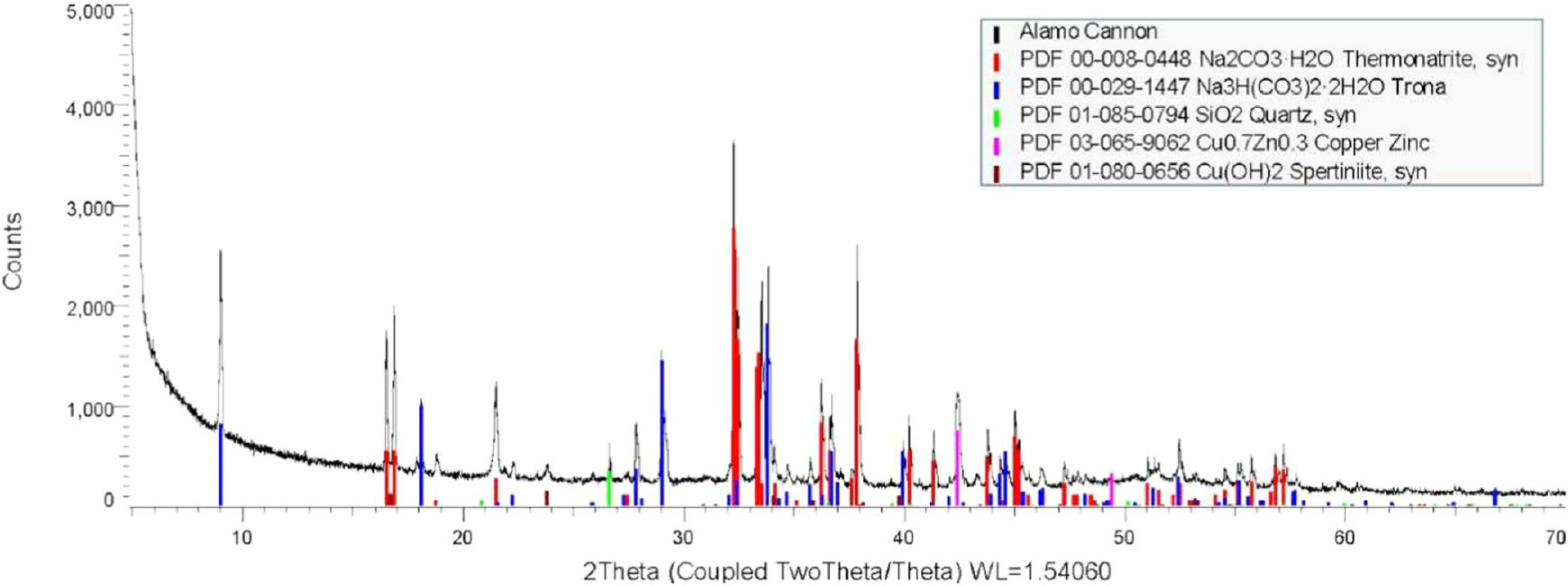
Data collected and analyzed via XRD to identify the components of the precipitate (analysis performed by the X-ray Diffraction Laboratory at the Department of Chemistry at Texas A&M University).

The presence of two sodium carbonate salts suggests that the powder is a precipitate from the chemicals used in the conservation process which remained inside the cannon’s matrix despite the boiling DI rinses. Residual sodium hydroxide can react with carbon dioxide in the air to form sodium carbonate and water

1 $$2\mathrm{NaOH}\;\left(\mathrm{aq}\right)+{\mathrm{CO}}_{2}\;\left(g\right)\to {\mathrm{Na}}_{2}{\mathrm{CO}}_{3}\;\left(\mathrm{aq}\right)+H_{2}O\;\left(l\right)$$

With carbonate ions thus present in solution, sodium carbonate variants would precipitate as the water evaporated during the drying process. Trona is one of the most common naturally occurring sodium carbonate variations in the world.^7^ It requires the presence of both sodium carbonate and bicarbonate, which is supplied by the equilibrium between the two anions in solution

2 $$2{\mathrm{HCO}}^{3-}\;\left(\mathrm{aq}\right)↔{\mathrm{CO}}_{3}^{2-}\;\left(\mathrm{aq}\right)+H_{2}O\;\left(l\right)+{\mathrm{CO}}_{2}\;\left(g\right)$$

In CO_3_^2–^ -dominant samples with a high pH, trona precipitates first, followed by thermonatrite.^8^ In such samples, thermonatrite is dominant when evaporation happens very quickly, and carbon dioxide is not allowed to dissolve into solution and buffer the solution. This may be the reason why thermonatrite is more prevalent than any other precipitate in both of our samples. Because the cannon is moved directly from the low concentration BTA solution to hot wax, then allowed to remain in the wax until all solvents have boiled out, solvent evaporation happens very quickly.

The powder was again analyzed using XRD in March 2023, after the cannon had been re- treated using sodium carbonate as an electrolyte. Again, thermonatrite was the most prevalent mineral followed by trona, then three minor precipitates: quartz (SiO), copper + zinc (Cu:0.7, Zn:0.3), and spertiniite. The new results supported the hypothesis that the electrolyte being used in conservation was remaining in the matrix and precipitating after the wax was used to seal the cannon. In this case, because the cannon was already being treated in a low concentration of sodium carbonate, reaction 1 would be unnecessary to reach a point in which thermonatrite and trona could easily precipitate.

Additionally, the basic environment caused the formation of spertiniite (Cu­(OH)_2_), a rare corrosion product which has been reported primarily in copper alloy artifacts cleaned with ammonia.^9^ This cannon has never been exposed to concentrated ammonia, however Cu­(OH)_2_ can also form alongside carbonates when copper alloys are treated with alkali hydroxides like NaOH or KOH.^10^ This may explain why the proportion of spertiniite was much higher in the sample taken after the cannon had completed treatment in NaOH when compared to the sample taken after the cannon was retreated with NaCO_3_ as an electrolyte.

We are concerned about this issue for two reasons. The first is the more immediate: the powder creates an aesthetic unsuitable for display at the Alamo Museum. Although the majority of the powder currently seems benign (ie. most of the precipitate has no metal component to indicate that its precipitation has damaged the cannon), it is visually unappealing. Visual appeal is a crucial component for artifacts that have such a central role in a museum display, as this cannon does at the Alamo.

Secondarily, there is a chance that while the trona and thermonatrite are not indicative of a current corrosion process they may lead to corrosion problems later. Indeed, chalconatronite (Na_2_Cu­(CO_3_)_2_·3H_2_O) is a known byproduct of corrosion caused by extended exposure of copper alloy artifacts to sodium carbonate and sodium sesquicarbonate (trona) through either conservation in sodium sesquicarbonate or carbonate solutions or through exposure to soils high in natural carbonate concentration.^9,11,12^ While the cannon has not yet shown signs of chalconatronite development, it was deemed important to prevent it by neutralizing the sodium carbonate issue before it progressed to damaging the bronze.

## Methods

In an attempt to replicate the issue, 12 small bronze ingots were cast for experimental use using Ancient Bronze Casting Grain purchased from Rio Grande Jewelry Supply, which contains 90% copper and 10% tin. They were subjected to 2 weeks of electrolytic reduction in 2% NaOH in deionized water at 5 A and 5 V. Following electrolytic reduction, the ingots were boiled in alternating deionized water rinses for five and a half hours, then soaked in 1% w/w benzotriazole in deionized water for a week and coated with microcrystalline wax to remove all volatile solvents. They were left for a month in a hot, damp location to encourage the migration of carbonates to the surface of the ingots. Despite our efforts, the ingots did not develop the same problem as the initial cannon. Because of pressing concern from the Alamo to address the issue and an unwillingness to experiment directly on the cannon due to the importance of the artifact, a procedure was developed to mimic the outward effects of the condition. Therefore, sodium carbonate was applied topically to the ingots to replicate the symptoms.

An acid was needed to neutralize the carbonates and bicarbonates that were precipitating on the cannon’s surface. By applying an acid, the carbonate would react to form carbon dioxide and water, effectively eliminating the original problem. However, the conjugate base of the acid, along with the sodium cations, could remain on the surface of the bronze in small quantities after rinsing, meaning they had to be chosen carefully. Acids were chosen on the basis of several known properties. Focus was put on acids which are already commonly used in archeological conservation, particularly the conservation of bronze and other copper alloys. These acids are easily accessible to our and other conservation laboratories, and are already known to produce minimal negative effects in archeological bronze. A short literature review produced citric, formic, and sulfuric acids as a starting point.^13–15^ In addition to these three, we generated a list of common acids and recorded their acid dissociation constant(s) (*K*_a_, *K*_a2_, etc.), molecular weight, and solubility of their sodium salt in water as seen in Table 2. We were looking for acids with a relatively high *K*_a_ to ensure that the acid would be strong enough to react with the sodium carbonate, had a low molecular weight which might encourage them to better penetrate the microcrystalline wax, and had a soluble sodium salt which would not create long-lasting problems that were harder to deal with than the original sodium carbonate salt. Additionally, the acids needed to be readily available and reasonably safe in low concentrations for both copper/copper alloys and for human handling. With these qualifications, we added acetic and phosphoric acid to the list.

**Table 2:** Relevant Properties of the Five Acids Chosen for the Experiment

acid	formula	sodium salt	*K*_a_	*K*_a2_	sodium salt solubility in water (20–25C) (g/100 mL)	MW acid (g/mol)
citric	C_6_H_8_O_7_	Na_3_C_6_H_5_O_7_	7.4 × 10^–4^ ^16^	1.7 × 10^–5^ ^16^	71 (dihydrate)^17^	192.124
formic	C_2_H_2_O	NaHCO_2_	1.7 × 10^–4^ ^18^	N/A	97.2 ^19^	46.03
sulfuric	H_2_SO_4_	Na_2_SO_4_	high	1.0 × 10^–2^ ^20^	28.1 ^21^	98.079
acetic	CH_3_CO_2_H	NaCH_3_CO_2_	1.75 × 10^–5^ ^22^	N/A	50.4 ^21^	60.052
phosphoric	H_3_PO_4_	Na_2_HPO_4_	6.9 × 10^–3^ ^21^	6.17 × 10^–8^ ^21^	11.8 ^21^	97.99

The acids were diluted with deionized water to create 5% v/v solutions (w/w for citric acid, which was added in powder form). Five percent v/v was chosen in alignment with common recommendations in the conservation field, which can be between 2 and 10%, depending on the acid and purpose.^13^ To maintain consistency for comparison, 5% was chosen for all acids. Using volume or weight percent rather than molarity or molality is standard procedure in the field to facilitate ease of calculation and mixture. This does lead to some variation in the quantity of acid molecules in each solution, with higher molecular weight acids containing less of the acid molecule per liter than lower molecular weight acids. The diluted acid was applied topically to the ingots with a cotton swab, then cleaned off with another cotton swab dipped in deionized water. This was meant to simulate the most practical way of applying the acid to the cannon: topical application with a swab or cloth, and wiping the acid away with another cloth soaked in deionized water. More intensive methods of application are impractical for an active museum setting.

Each of the acid solutions was applied three times to the ingots. The second application occurred 1 week after the first, and the third application occurred 6 weeks after the second. This timing allowed time for precipitate to reform on the surface after treatment and for extended observation. The ingots were observed a day after application, a week after application, and in the case of the second and third applications, several weeks after application. At each observation, photos were taken to document the surface appearance and pH testing was done using pH strips and deionized water to read the surface of the ingot. Additionally, optical microscopy was used to determine whether there was precipitate which was not visible to the naked eye, and to get a more accurate image of the color of the precipitates.

## Results

Immediately after application of the acid, the carbonate on each of the experimental ingots produced bubbles and gas, indicating that the acids were reacting with the carbonate as expected to generate carbon dioxide and water, and new sodium salts. However, carbonate blooms resurfaced on all ingots except those treated with phosphoric and acetic acids within the first 2 days of the initial acid application. The blooms were identified as continued carbonate precipitation based on their white color and extremely basic pH. The pH for ingots treated with phosphoric acid and one ingot treated with acetic acid decreased from 10 to 9 after the first application, while the others stayed at 10. By 1 week after the acid application, carbonate blooms were also visible on ingots treated with phosphoric and acetic acids.

The pH of all experimental ingots decreased after the second application of the acid. Those treated with citric acid remained slightly higher than neutral while the remaining experimental ingots were neutral (or acidic, in the case of sulfuric acid). However, ingots treated with acetic acid increased in pH over the next 7 weeks and again reached a pH of 8–9, an increase not seen in other experimental groups. Precipitate and/or corrosion bloomed again on many of the ingots after 7 weeks. The most obvious blooms were on the sulfuric acid-treated ingots, which had yellowish blooms indicative of sulfate salts. Combined with the recorded low pH of these two ingots, it seems probable that the precipitating salt is not sodium carbonate but rather a sulfate salt, possibly sodium sulfate combined with residual acid to account for the low pH. Salt formation was also visible on ingots treated with acetic acid, which had a high pH indicating possible carbonate reprecipitation. No extensive powder precipitated on ingots treated with citric or phosphoric acids. However, after 6 weeks it also became evident that corrosion was occurring in ingots treated with acetic and citric acids. The corrosion was evident in green crystals on the surface of the ingots, indicating that some of the copper had been incorporated into corrosion products. Crystal formation was widespread in one acetic acid-treated sample and localized in the other as well as both citric acid-treated samples.

After the discovery of the corrosion products, the ingots were treated a third time to determine whether the corrosion would return or become an issue in other treatment groups. As shown in Figure 5, the week after the third treatment, there was extensive corrosion visible on both ingots treated with phosphoric acid. No other acids showed the green corrosion after only 1 week.

**Figure 5:**
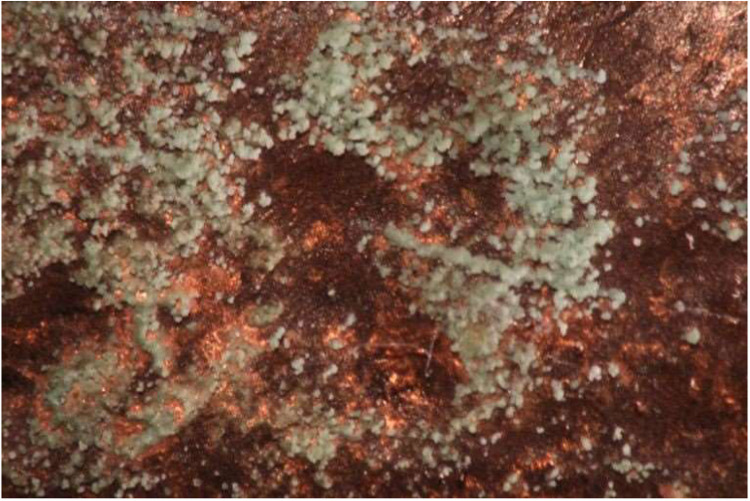
Ingot 5, showing the extensive green corrosion product appearing after the third application on the surface of ingots treated with phosphoric acid.

As shown in Figure 6, the ingots treated with sulfuric acid continued to show an acidic, yellow precipitate, while Figure 7 shows the carbonate blooms which reappeared on the control ingots. The control ingots were the only group which retained a pH of 9–10, indicating that carbonate remained on the surface of the controls but none of the experimental groups. The other ingots were recorded at pHs of 4 (sulfuric acid), 6–6.5 (citric acid), 7 (formic and phosphoric acids), and 8 (acetic acid). White, slightly basic blooms also appeared on the ingots treated with acetic acid, suggesting a continued return of the sodium carbonate, most likely mixed with sodium acetate given the slightly lowered pH.

**Figure 6:**
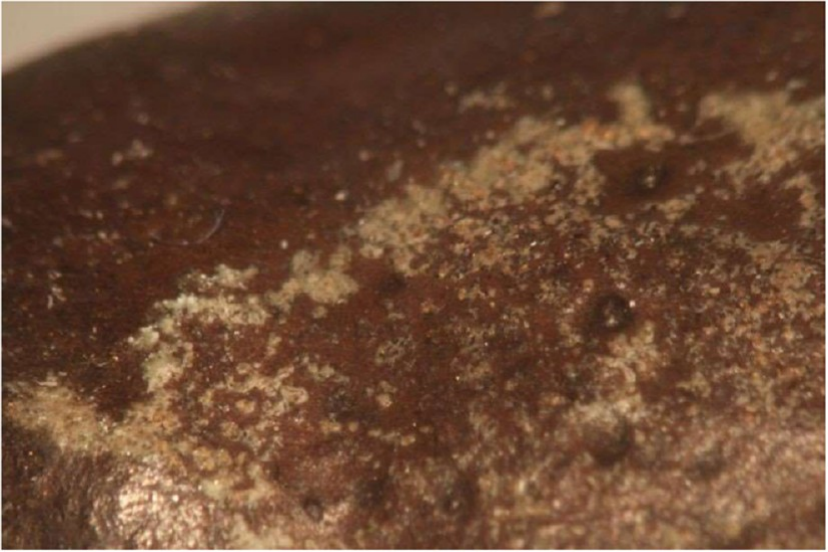
Ingot 9, showing an example of the yellow precipitate on the surface of ingots treated with sulfuric acid.

**Figure 7:**
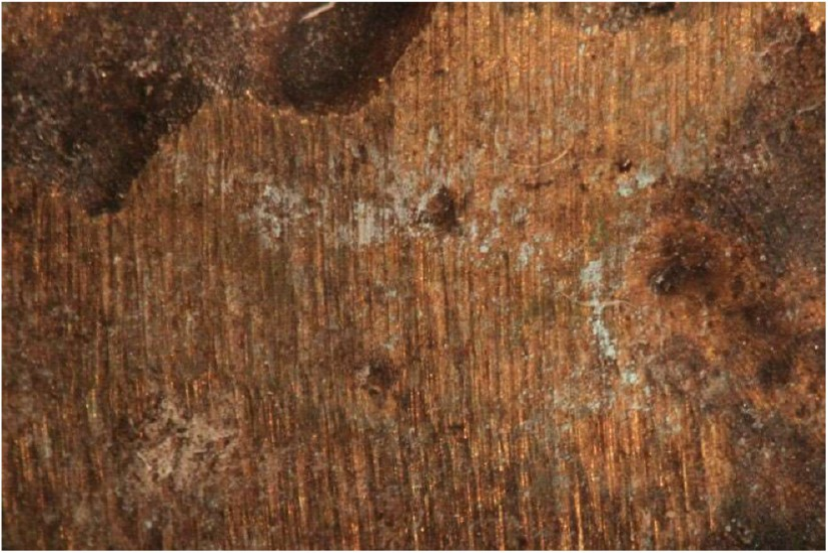
White carbonate blooms reappeared on the surface of the control ingots after the third application.

A month after the third treatment, the ingots appeared much the same as they had after 1 week. The samples treated with phosphoric and sulfuric acids both showed some alterations to the surface appearance of the wax and underlying metal, suggesting that the acids may be reacting with the samples themselves. In addition to the phosphoric acid samples, Figure 8 demonstrates that the bronze treated with acetic acid also showed some green corrosion, although more limited than after the second application, in addition to the widespread white blooms.

**Figure 8:**
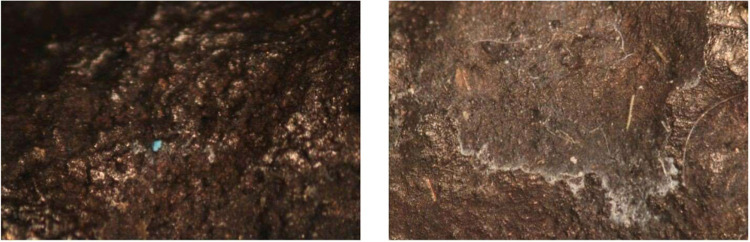
Microscopy photographs of ingot 7 (left) showing green corrosion and 8 (right) showing white precipitate; examples of precipitate visible post-treatment on ingots treated with acetic acid.

Figures 9 and 10 show that both the samples treated in citric acid and those treated with formic acid appeared stable, with limited white crystalline precipitate visible under the optical microscope but not noticeable unaided. Three months after application, there was evidence of a small amount of green corrosion on both sets of samples. This was also visible only under an optical microscope.

**Figure 9:**
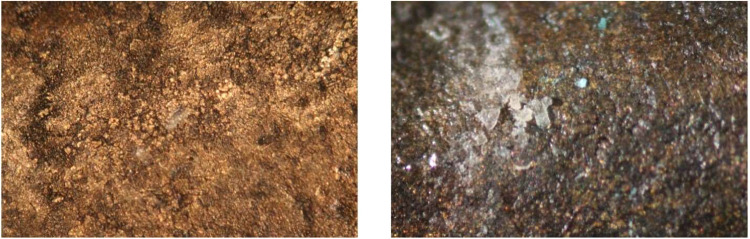
Minor white precipitation on the surface of ingot 1, treated with citric acid (left) and both white precipitate and green corrosion on the surface of ingot 2 (right).

**Figure 10:**
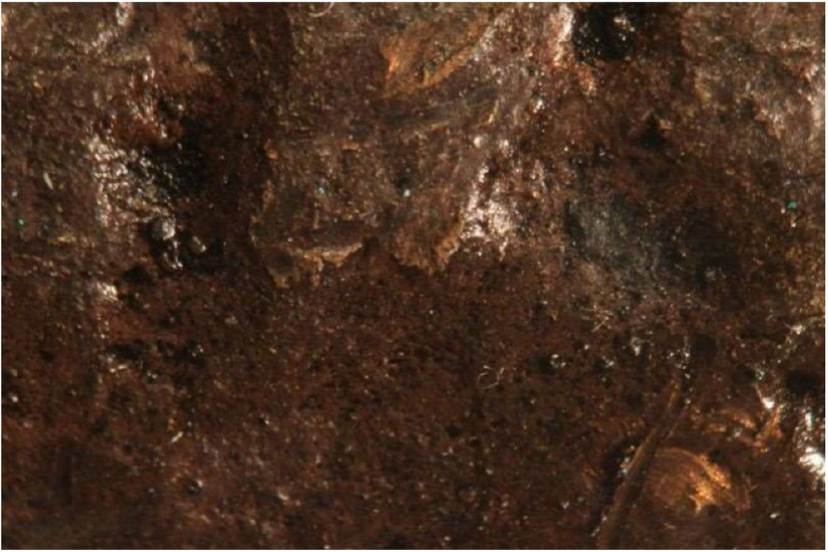
Surface of ingot 3, treated with formic acid. Some minor green and white corrosion is visible on the surface.

Three months after the third application, the control ingots also began to show signs of copper corrosion, as seen in Figure 11. This partially replaced the previously visible white precipitate. Eighteen months after the third application, the ingots had not changed in appearance, and corrosion products on the surface of the formic and citric acid ingots had not grown (Table 3).

**Figure 11:**
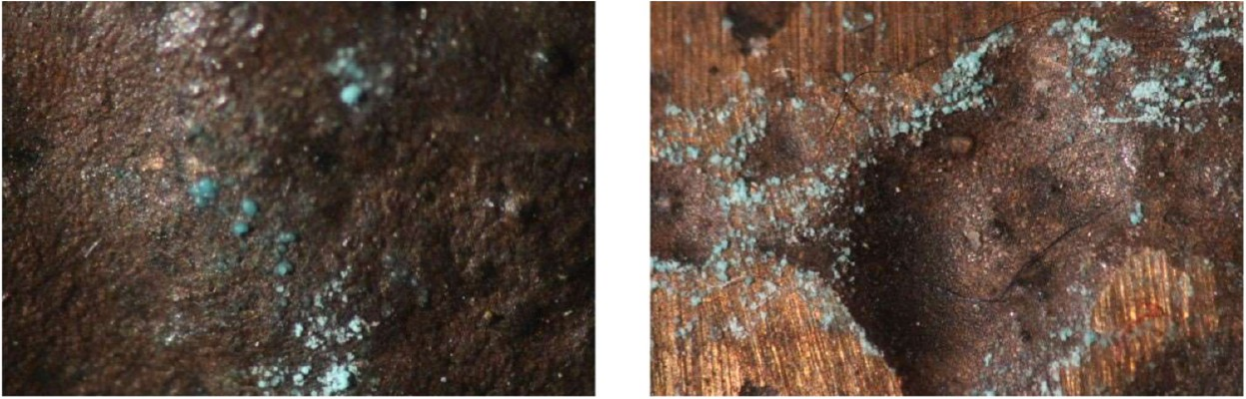
Copper corrosion is visible on the surface of ingot 11 (left) and ingot 12 (right), both control ingots treated only with DI water.

**Table 3:** Summary of the Results of the Acids

group	final pH	presence of corrosion	first appearance of corrosion	presence of carbonate	final appearance of carbonate	other notes
citric	6–6.5	localized	6 weeks after second application	no	after first application	localized salt precipitate
formic	7	localized	3 months after final application	no	after first application	localized salt precipitate
sulfuric	4	none	–	no	after first application	sulfate salt precipitation after second application, altered surface appearance
acetic	8	extensive	6 weeks after second application	possible	after second application	basic white precipitate after third application may be carbonate
phosphoric	7	extensive	1 week after final application	no	after first application	altered surface and wax appearance
control	9–10	extensive	3 months after final application	yes	continued to end of experiment	precipitate slowly disappeared as corrosion formed

## The Cannon

Following the completion of the proxy experiment and approval by stakeholders, our team visited the Alamo to begin the application of formic acid to the cannon. A solution of 5% v/v formic acid in deionized water was applied to the surface of the cannon using cotton pads. The powder fizzed on application of the acid, indicating the formation of carbon dioxide. Acid was applied until gas generation ceased and the surface was clear of white powder. The solution was applied to the inside of the bore using a tennis ball on the end of a long pole.

After formic acid application, cotton pads were soaked in deionized water and used to remove remaining acid residue from the cannon. A sponge replaced the tennis ball for the cleaning of the bore. Treated sections were rinsed in this way three to four times before the cannon was dried and the exhibit glass was replaced.

Figure 12 shows the initial results, which saw the cannon entirely cleared of carbonate precipitation. It remained clean for a few weeks, after which the precipitate reappeared. A second application of 5% formic acid was required and was applied six months after the first application. It was again cleaned from the surface thoroughly using deionized water. This was expected, given that the proxy experiments also required multiple applications to clear the precipitate from the surface. Following the second application, the precipitate reformed more slowly and in lower quantities. Figure 13 shows the cannon approximately two months after the second application, and eight months after initial application. Further applications are planned until the precipitate does not return. During this time, the Alamo staff also observed that green corrosion products reappeared on the cannon. It is not currently clear whether these remain from before formic acid application or whether it is new, but it is being closely monitored. It may be the result of formic acid application, as shown in the proxies. However, as the other ingots also exhibited extensive corrosion and there was some corrosion present before treatment, it is likely that some portion of it is the result of the water or an effect of previously applied citric acid.

**Figure 12:**
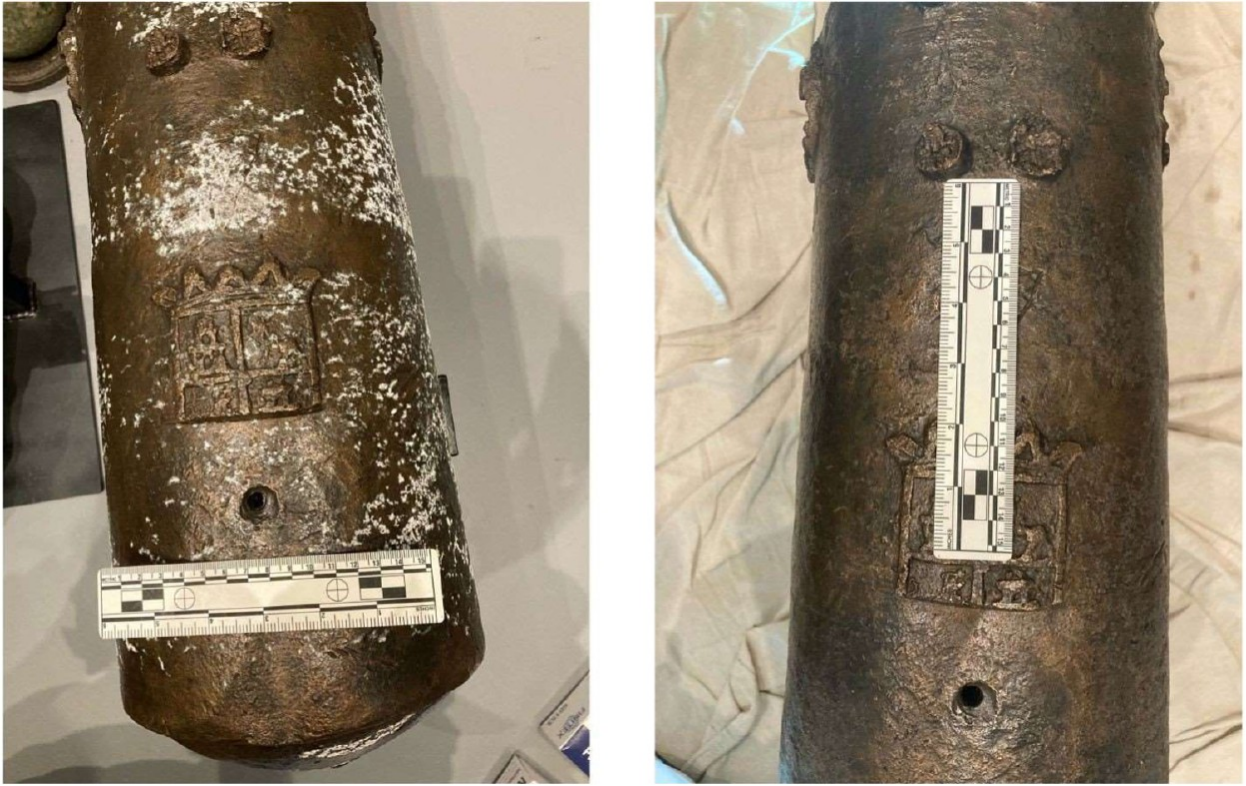
Photos of the cannon before (left) and immediately following (right) application of formic acid to remove the hydrated carbonate precipitation.

**Figure 13:**
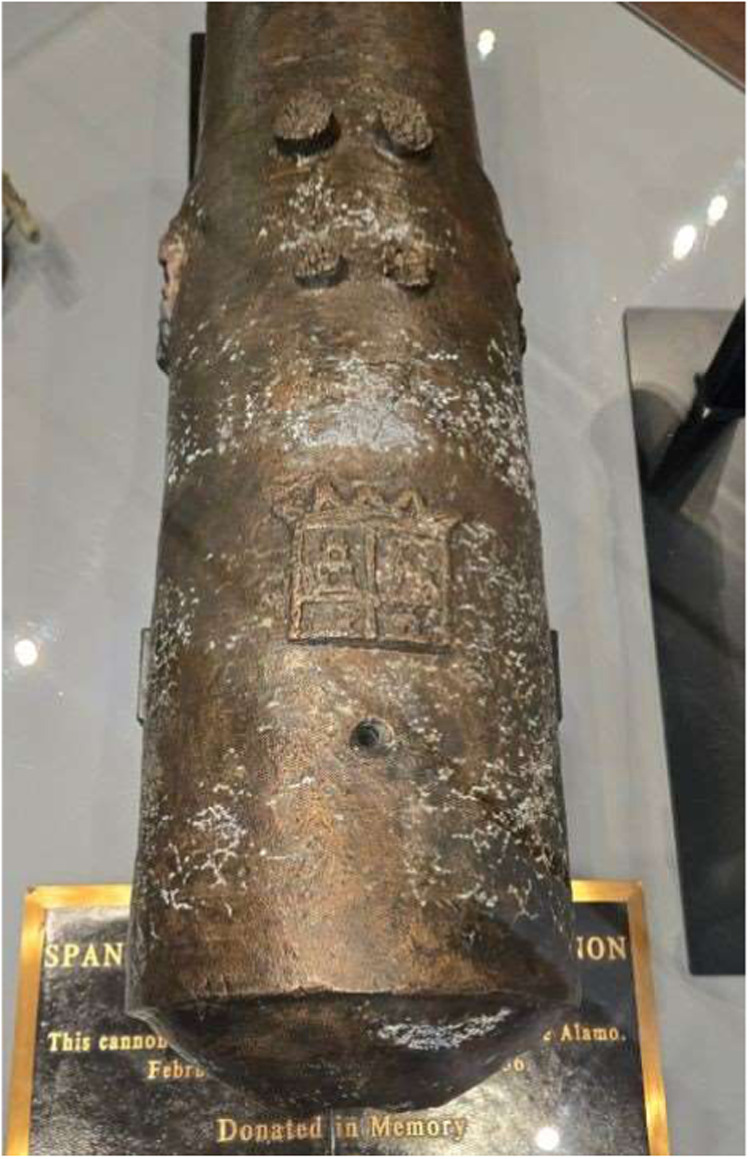
Cannon two months after the second application (eight months after initial application).

## Discussion

Based on initial experimentation, several conclusions were drawn about how to go forward with the treatment of the cannon. It was immediately clear that some treatment was required beyond the application of deionized water if the problem was to be resolved with any efficiency. Within just a few days of each application, carbonate reprecipitated on the surface of the experimental ingots, indicating that a DI wash was not sufficient to resolve the issue, particularly considering that the precipitate on the cannon seems to be coming from within the pores of the metal. Because the DI water was incapable of addressing the issue on the experimental ingots, it was extremely unlikely that it would be able to address the issue on the real cannon, where the issue was much more complex. Furthermore, the presence of copper corrosion on the control ingots indicated that the bronze would corrode even without the presence of acid. Indeed, it was more widespread than corrosion in either the citric or formic acid experimental groups. This may be evidence that the proposed concerns of sodium carbonate corroding the surface of the artifact to form chalconatronite are valid, since it could form through reactions between the copper and the carbonates.^11^ Regardless, it demonstrated the necessity of treating the cannon, despite possible negative effects of the acid application, since allowing the carbonate mixture to remain in the cannon would also be cause corrosion issues.

Additionally, one application of any treatment would be insufficient to address the problem, as the carbonate reprecipitated on the surface of all experimental ingots after only one application. Sulfuric acid was ruled out early in the process because of the appearance of extensive sulfate precipitate. Its low pH had a negative effect on the wax and underlying metal, and it is likely to have a negative long-term effect on the artifact’s stability if applied topically, as DI rinses did not seem to adequately remove it. Other conservators have also noted base metal deterioration after sulfuric acid pickling.^14^ Additionally, the application of sulfuric acid resulted in unsightly yellow surface precipitation which was difficult to remove and reappeared after rinsing. Phosphoric acid was removed from consideration for similar reasons, having a negative effect on the wax and underlying metal. It also resulted in widespread surface corrosion of the metal after the third application. Phosphoric acid has previously been noted to encourage corrosion in copper and copper alloys.^23^ Although there is some concern over formic acid being a source of formates and leading to copper formate corrosion,^24^ this experiment did not find it to be a worse source of corrosion than other options. Results were similar to those of citric acid, which is also known to corrode copper but is still used in copper-alloy conservation when necessary.^25^ Indeed, only sulfuric acid showed no signs of copper corrosion, but it did show uneven coloring and base metal deterioration even without the generation of green crystals.

Potential treatments were evaluated based on the American Institute for Conservation (AIC) Code of Ethics for Conservation of Historic and Artistic Works, particularly sections VI and VIII, which specify that conservators ought to choose methods which, to the best of their ability and knowledge, will not lead to long-term damage to the artifact and will prevent future degradation.^26^ It was clear that allowing the precipitate to remain on the cannon or attempting less interventive treatment through the application of deionized water to remove the precipitate would violate section VIII, as results indicated that it would cause later corrosion. Low concentration formic acid was deemed to be the treatment most in keeping with section VI, as it caused the least observable long-term damage. Effectiveness was also evaluated, although all acids resulted in a lower pH and less observable carbonate after treatment. The predicted reaction mechanism for all acids is the following, where R is the conjugate base of the acid. For polyprotic acids, the mechanism is expected to be the same but the ratio of acid molecules to carbonate molecules would vary:

3 $$Na_{2}CO_{3}+2\mathrm{HR}\to 2H_{2}O+CO_{2}+2\mathrm{NaR}\;\left(\mathrm{aq}\right)$$

While the reactions between the carbonate and the acids were expected to be similar, bronze is known to corrode at different rates depending on which acid(s) it is exposed and depending on the composition of the bronze.^27^ Bronzes used in cultural heritage are particularly variable in their composition, and may exhibit different metal proportions across the same artifact, resulting in uneven corrosion. Conservators have long noted the ability of both buffered and unbuffered citric acid to etch bronze artifacts during treatment,^13^ and corrosion rates have been calculated for bronze when exposed to phosphoric acid since the 1930s.^28^ Heritage professionals have found that sulfuric acid corrodes bronzes differently based on their tin content, with higher tin contents forming more stable patinas to protect against low concentration acids.^29^ While research has been done to examine the effects of acids on metals, especially modern alloys used in engineering, little work has quantitatively or qualitatively compared the effects of the acids to each other. Atmospheric scientists using proxies have found formic acid vapors to be more corrosive to bronze than acetic acid, and implicate chlorides, carbon dioxide, and organic acids in the corrosion processes.^30^

Formic acid was found to be less corrosive than acetic acid in this study, resulting in only microscopic corrosion which did not appear to worsen significantly over the year and a half observation period. This may be because sodium formate is more soluble in water than sodium acetate, as shown in Table 2, leading to a more thorough cleaning when deionized water was used to swab the surface after acid application. Evidence is lent to this theory by the fact that citric acid, which has the second highest solubility salt (sodium citrate), also had a lower prevalence of corrosion than the other experimental acids.

After analyzing the results, a low concentration formic acid was chosen to treat the cannon, and is being considered preliminarily successful after 8 months. It removed the carbonate precipitation for several weeks before reapplication was required, and the second application removed the precipitate for longer and resulted in a smaller quantity returning. We expect that reapplication will be required less and less frequently going forward, as more of the carbonate is cleared from the cannon. Based on 18 months of observation on the experimental ingots, we also do not expect there to be significant negative effects on the cannon. It is significantly more effective at keeping the precipitate at bay than the previous solutions, which were deionized water (effective for 2–3 days) and low concentration citric acid (effective for a month or less after multiple applications).

It is important to note how common corrosion was as a side effect of the acid application, even at low concentrations and after using DI water to clean the surface after application. Corrosion appeared on at least four of the five experimental groups at different points in the experimental process: citric acid, formic acid, phosphoric acid, and acetic acid. It also appeared on the control group. Phosphoric and acetic acid each produced instances of widespread corrosion visible to the naked eye. This indicates that even low concentration acids and bases are able to penetrate microcrystalline wax, since the acid was applied to the surface of the ingot postwax treatment, but there was corrosion of the copper in the metal. While the surface of the phosphoric acid samples were visibly altered by the acid, the acetic and citric acid samples appeared the same as they had previously, indicating that not only were some of the acids reacting with the wax but they were able to migrate in and out of the wax without destroying it entirely.

Microcrystalline waxes are relatively high molecular weight (580–700) hydrocarbons made from petroleum byproducts,^31^ which are generally considered water resistant. They are most often used to protect artifacts from the elements due to their nonpolar nature, which prevents the diffusion of polar molecules through the surface. The ability of the acids to penetrate the wax has implications for the effective protective nature of microcrystalline wax for heritage conservation, a topic which has been previously raised by other conservators.^32,33^ It has been suggested that wax is not an especially effective protective coating for more than a few years, particularly when used outdoors, and this research supports such a conclusion and encourages further research into the effectiveness of surface coatings for artifacts.

## Conclusions

While often used to conserve copper-alloy artifacts, especially those exposed to exterior or underwater environments, electrolytic reduction has the potential to cause conservation concerns in bronze artifacts if the electrolyte is unable to exit the object completely. This appears to have been the case in the bronze 4-pounder cannon on display in the Alamo, on which carbonate-based precipitates appeared soon after conservation was completed. A proxy experiment was conducted to determine the best and most practical way to treat the gun, comparing the effectiveness of five dilute acids to treat carbonate blooms. Of the five acids, phosphoric, acetic, formic, and citric all showed signs of causing copper corrosion in the form of a green crystalline structure appearing on the surface of the proxy artifacts. Deionized water alone also generated extensive copper corrosion. Sulfuric acid caused significant deterioration of the wax and underlying metal, and resulted in an acidic yellow precipitate that was unsightly and difficult to remove. Formic acid showed the least recurring carbonate precipitation and a relatively low corrosion potential compared to other options, including the control group.

Based on these results, a low concentration formic acid was chosen to treat the cannon. It was effective in removing the precipitate from the surface, although reapplication was necessary after a few months. It is expected that the period between reapplications will become longer as more of the carbonate reacts with the acid. Additionally, the formic acid does not appear to be doing significant damage to the matrix of the cannon after eight months of observation. Some green corrosion product is present on the cannon and is being monitored to ensure it does not grow, but it is unclear whether this is the result of the carbonate, the acid, or a mix of the two.

## Supplementary Material





## Notes

Description of XRD specifications, experimental parameters (PDF)
Ingot Statistics (XLSX)
