# Supplementary material for: Addressing the Precipitation of Hydrated Carbonates on a Bronze Cannon from the Alamo
Source: ACS Omega. 2025 Aug 19;10(34):38686–95. doi: 10.1021/acsomega.5c03779 (PMC12409588; doi:10.1021/acsomega.5c03779)
Supplement: Supplementary file 1 [file ao5c03779_si_001.pdf]

Addressing the precipitation of hydrated carbonates on a bronze cannon from the alamo

*Kimberly L. Breyfogle and Christopher Dostal**

Department of Anthropology, Texas A&M University, College Station, Texas, United States of America
*Corresponding author
Email: dostalc@tamu.edu

XRD Instrument Specifications as provided by the X-Ray Diffraction Laboratory and Texas A&M University.

The sample was placed in the sample holder of a two circle goniometer, enclosed in a radiation safety enclosure. The X-ray source was a 1kW Cu X-ray tube, maintained at an operating current of 40 kV and 25 mA. The X-ray optics was the standard Bragg-Brentano para-focusing mode with the X-ray diverging from a DS slit (1mm) at the tube to strike the sample and then converging at a position sensitive X-ray Detector (Lynx-Eye, Bruker-AXS). The two-circle 218mm diameter θ-θ goniometer was computer controlled with independent stepper motors and optical encoders for the θ circle with the smallest angular step size of 0.0001^{o} 2θ. The software suit for data collection and evaluation is windows based. Data collection is automated COMMANDER program by employing a DQL file. Data is analyzed by the program EVA.

Table S1: Experimental Parameters

Wavelength	1.54060Å
Detector	PSD (Lynx-Eye Bruker AXS)
Anti-scatter Slit	12.530mm
Divergence Slit	1.00mm
Anti-air-scatter	Knife edge
Scan type	Coupled theta/2theta
Goniometer radius	217.5mm
Start	4.0
End	70.0
Step Size	0.015
Total Scan Time	60mins

The relevant statistics for the ingots before, during, and after treatment can be found in the Excel spreadsheet Table S2.
